# Supplementary material for: Cellular automaton decoders for topological quantum codes with noisy measurements and beyond
Source: Sci Rep. 2021 Jan 21;11:2027. doi: 10.1038/s41598-021-81138-2 (PMC7820354; doi:10.1038/s41598-021-81138-2)
Supplement: Supplementary file 1 — Supplementary Information [file 41598_2021_81138_MOESM1_ESM.pdf]

Supplementary Information for "Cellular automaton decoders for topological quantum codes with noisy measurements and beyond"

Michael Vasmer,[1, 2, 3, *] Dan E. Browne,[1] and Aleksander Kubica[2, 3, †]

[1] *Department of Physics and Astronomy, University College London, Gower Street, London, WC1E 6BT*
[2] *Perimeter Institute for Theoretical Physics, Waterloo, ON N2L 2Y5, Canada*
[3] *Institute for Quantum Computing, University of Waterloo, Waterloo, ON N2L 3G1, Canada*

(Dated: December 23, 2020)

SUPPLEMENTARY NOTE 1

In this note, we fill in the details of the proof of the non-zero threshold of the sweep decoder (Theorem 1). The proof strategy is essentially the same as in Ref. [1]. First, we describe a decomposition of errors into chunks, which aids our analysis. Then, we show that the sweep decoder successfully corrects chunks of the error, up to a certain size. Finally, we find that the probability of the error containing chunks that cause the decoder to fail is exponentially suppressed in the linear size of the lattice.

Chunk decomposition of the error

We begin by defining the diameter of a subset of vertices, $U \subseteq \mathcal{L}_0$, to be the maximal distance between any two vertices in U, i.e. $\mathrm{diam}(U) = \max_{u,v\in U} d\,(u,v)$, where $d\,(u,v)$ is the length of the shortest path linking u and v in $\mathcal{L}$. Let $\epsilon \subset \mathcal{L}_2$ denote an error contained in a local region of $\mathcal{L}$. We now define a decomposition of errors into chunks, following [2]. A level-0 chunk, $E^{[0]}$ is an element of ϵ and a level-n chunk is a defined recursively to be the disjoint union of two level-$(n-1)$ chunks $E_1^{[n-1]}$ and $E_2^{[n-1]}$, where $\mathrm{diam}(E^{[n]}) \leq Q^n/2$ for some constant Q. The level-n error $E_n \subseteq \epsilon$ is defined to be the union of all level-n chunks. We note that

$$\epsilon = E_0 \supseteq \ldots \supseteq E_m \supsetneq E_{m+1} = \emptyset. \tag{1}$$

Therefore, we can decompose the error into $F_n = E_n \setminus E_{n+1}$, for $n \in \{0,\ldots,m\}$ as follows

$$\epsilon = F_0 \sqcup \ldots \sqcup F_m, \tag{2}$$

where $A \sqcup B$ denotes the disjoint union of A and B. We say that $M \subseteq \epsilon$ is an l-connected component of ϵ if, for any $M_1, M_2 \neq \emptyset$, if $M = M_1 \sqcup M_2$ then $d(M_1, M_2) \leq l$. We need the following lemma, which concerns the size and separation of connected components of F_i.

Lemma 1. *(Connected Components [2]). Let ϵ be an error with disjoint decomposition $\epsilon = F_0 \sqcup \ldots \sqcup F_m$ and let $Q \geq 6$ be a constant. Suppose that $M \subseteq \epsilon$ is a Q^n-connected component of F_n. Then,* $\mathrm{diam}(M) \leq Q^n$ *and* $d\,(M, E_n \setminus M) > Q^{n+1}/3$.

Lemma 1 gives us both an upper bound on the size of any Q^n-connected component of the error and a lower bound on the separation of the Q^n-connected component from the rest of the error. For a proof, see [1, 2].

Correction of high-level chunks

Lemma 2. *Let $\epsilon \subset \mathcal{L}_2$ be an error with disjoint decomposition $\epsilon = F_0 \sqcup F_1 \ldots F_{m^*-1}$. Choose constants $Q = 6|\Omega| c_P c_D^8$ and $m^* = \lceil \log_Q(L/2c_D^8) \rceil$, where L is the linear lattice size, Ω is the set of sweep directions, $c_D = 2$ and $c_P = 1$. Suppose we apply the sweep decoder with $T_{max} = c_P c_D^8 Q^{m^*}$. Then, ϵ is corrected, i.e. the product of ϵ and the correction returned by the sweep decoder is a stabilizer.*

---

* mvasmer@perimeterinstitute.ca

† akubica@perimeterinstitute.ca

The proof of the above Lemma is essentially the same as the proof of Lemma 3 in [1], albeit with adjusted constants. We briefly sketch the proof here, for completeness.

Let M be a Q^n-connected component of F_n. We chose the constant $m^* = \lceil \log_Q(L/2c_D^8) \rceil$ such that $\mathcal{R}(M)$ is contained in a local region of $\mathcal{L}$. One can verify that $\mathrm{diam}(\mathcal{R}(U)) \leq c_D \,\mathrm{diam}(U)$ for all $U \subseteq \mathcal{L}_0$ and $\vec{\omega} \in \Omega$, with $c_D = 2$. Therefore

$$\mathrm{diam}(\mathcal{R}(M)) \leq c_D^8 \,\mathrm{diam}(M) \leq c_D^8 Q^n < c_D^8 Q^{m^*} \leq \frac{L}{2} \tag{3}$$

By the removal property of the sweep rule, $\sigma = \partial M$ will be removed in $|\Omega| \times T^*$ time steps, where T^* is

$$T^* = \max_{\vec{\omega} \in \Omega} \max_{(\inf \mathcal{R}(\sigma) \updownarrow \sup \mathcal{R}(\sigma))} |(\inf \mathcal{R}(\sigma) \updownarrow \sup \mathcal{R}(\sigma))|. \tag{4}$$

We use the properties of the rhombic dodecahedral lattice to bound T^*. For any two vertices $u, v \in \mathcal{L}_0$, and any sweep direction $\vec{\omega} \in \Omega$, $\max_{(u \updownarrow v)} |(u \updownarrow v)| \leq c_P \times d(u,v)$, with $c_P = 1$. Therefore, we can upper-bound T^*:

$$T^* \leq |\Omega| c_P \,\mathrm{diam}(\mathcal{R}(M)) \leq c_P c_D^8 Q^n. \tag{5}$$

We choose the constant $Q = 6|\Omega| c_P c_D^8$ such that M is removed independently from $E_i \setminus M$. This follows from Lemma 1 and the propagation property of the rule. Finally, because $\mathcal{R}(M)$ is contained in a local region of $\mathcal{L}$, the product of M and its correction implements a trivial logical operator. Given the above, an inductive argument shows that all chunks up to level-m^* are corrected by the sweep decoder.

Probability of high-level chunks

The only remaining step in the proof of Theorem 1 is to show that the probability of the error ϵ containing level-m^* chunks is exponentially suppressed in the size of the lattice. This can be accomplished using a percolation theory argument, as explained in [1]. The outcome is the following bound on the probability of ϵ containing a level-n (or higher) chunk:

$$\Pr[\epsilon \text{ contains a level-}n \text{ chunk}] \leq \frac{|\mathcal{L}_0|}{\lambda^2} \left(\frac{p}{p_{\mathrm{th}}}\right)^{2^n}, \tag{6}$$

where $\lambda = (2Q)^3 c_B$. To specify c_B, we define a discrete ball of radius r centred at a vertex v, $B_v(r)$, to be the set of all lattice elements a distance smaller than r from v. The constant $c_B = 8$ is set by the fact that for any ball $B_v(R)$ contained within a finite region of $\mathcal{L}^\infty$, there exists a cover

$$\bigcup_{u \in U} B_u(r) \supset B_v(R), \tag{7}$$

consisting of balls of radius $r < R$ index by $U \subset \mathcal{L}^\infty$, such that

$$|U| \leq c_B (R/r)^3. \tag{8}$$

The threshold error probability p_{th} is

$$p_{\mathrm{th}} = \left(\lambda^2 \max_{v \in \Delta_0(\mathcal{L})} |\,\mathrm{St}_2(v)|\right)^{-1}, \tag{9}$$

where the 2-star of v, $\mathrm{St}_2(v) = \{f \in \mathcal{L}_2 : v \in f\}$. If we substitute $m^* = \lceil \log_Q(L/2c_D^8) \rceil$ into Eq. (6), we obtain:

$$\Pr[\epsilon \text{ contains a level-}m^* \text{ chunk}] \leq \frac{|\mathcal{L}_0|}{\lambda^2} \left(\frac{p}{p_{\mathrm{th}}}\right)^{\beta_1 L^{\beta_2}}, \tag{10}$$

where $\beta_1 = 1/(2c_D^8)^{\beta_1}$ and $\beta_2 = \log_Q 2$. As $|\mathcal{L}_0| = O(L^3)$, the probability of the error containing a level-m^* (or higher) chunk is $O\left((p/p_{\mathrm{th}})^{\beta_1 L^{\beta_2}}\right)$. □

For rhombic dodecahedral lattices, the value of the error threshold given by Eq. (9) is $p_{\mathrm{th}} \approx 10^{-30}$. This is many orders of magnitude smaller than the value we observe in simulations of $p_{\mathrm{th}} \approx 21.5\%$, which underlies the importance of using numerical simulations to estimate the error threshold of a decoder.

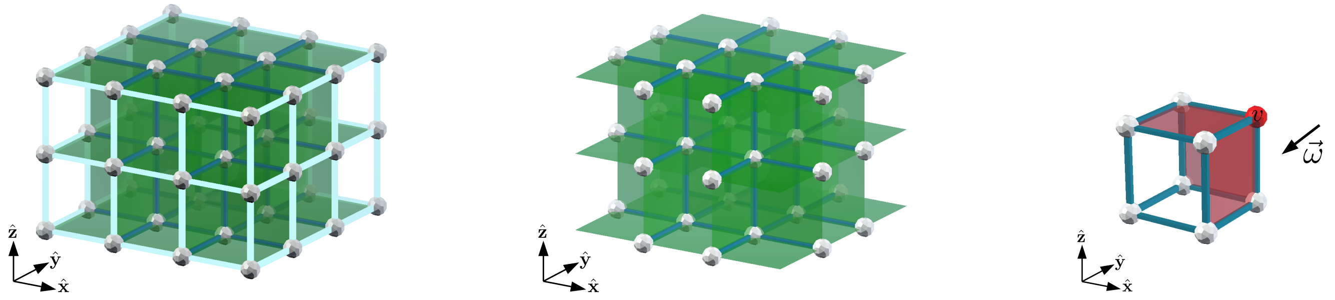

(a) (b) (c)

Supplementary Figure 1. (a) Constructing the $L = 3$ cubic toric code lattice. We illustrate how the lattice (dark blue edges and green faces) is a sublattice of the infinite cubic lattice (represented by light blue edges). The $L = 3$ cubic toric code lattice. We show all the vertices, edges and faces of the lattice. In particular, we note that some faces on the boundary have only two or three edges. (c) The action of the sweep rule at the vertices of the cubic lattice. The sweep direction $\vec{\omega} = -(1, 1, 1)$ points from the red vertex v into the centre of the cube. The shaded red faces are the faces that can be returned by the rule from v.

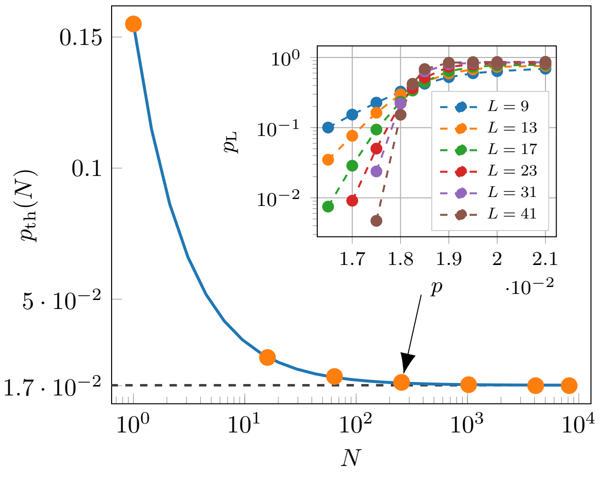

Supplementary Figure 2. Sustainable threshold of the sweep decoder applied to the toric code on the cubic lattice (with boundaries). We plot $p_{\text{th}}(N)$, the error threshold as a function of the number N of error correction cycles, for an error model with $\alpha = q/p = 1$. The inset shows the data for $N = 2^{10}$, where we use 10^4 Monte Carlo samples for each point. We observe a sustainable threshold of $p_{\text{sus}} \approx 1.7\%$, with $\gamma = 0.92$ (see Eq. (10) for the fit).

SUPPLEMENTARY NOTE 2

We applied the sweep decoder to toric codes defined on cubic lattices with open and periodic boundary conditions. For the case with open boundaries, we consider a family of toric codes constructed from the infinite cubic lattice. To construct a toric code with code distance L and one encoded qubit, we take a $L \times L \times (L-1)$ sublattice of the infinite cubic lattice with vertices at integer coordinates $(x, y, z) \in [0, L] \times [0, L] \times [0, L-1]$. Then we associate qubits (X stabilizer generators) with all faces (edges) except those in the $x = 0, L$ or $y = 0, L$ planes. Supplementary Figure 1 illustrates the construction of the $L = 3$ lattice. We use eight sweep directions $\Omega = \{(\pm 1, \pm 1, \pm 1)\}$, where each sweep direction points into the centre of the cubes. Supplementary Figure 1c explains how the rule works for one of the sweep directions (the rest are the same by symmetry).

We simulated the performance of the decoder for an error model with equal phase-flip and measurement error probabilities (i.e. $\alpha = 1$ in Definition 2). We observe a sustainable error threshold of $p_{\text{sus}} \approx 1.7\%$ for toric codes defined on lattices with and without boundaries; see Supplementary Figure 2. We find that the optimal decoder parameters were essentially the same as those we described for the rhombic dodecahedral lattice (see Results of the main text).

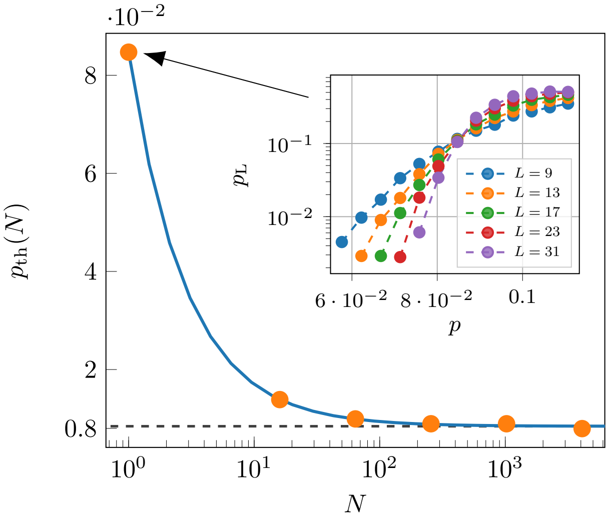

Supplementary Figure 3. Sustainable threshold of the sweep decoder applied to the toric code on the rhombic dodecahedral lattice (with boundaries) against correlated errors. We plot $p_{\rm th}(N)$, the error threshold as a function of the number of error correction cycles. The inset shows the data for $N = 1$, where we use 10^4 Monte Carlo samples for each point. We observe a sustainable threshold of $p_{\rm sus} \approx 0.8\%$, with $\gamma = 0.95$ (see Eq. (10) for the fit).

SUPPLEMENTARY NOTE 3

We ran simulations to estimate the performance of the sweep decoder against correlated noise for rhombic dodecahedral lattices with boundaries. We used a simple error model where, at each time step, every pair of neighbouring qubits experiences an error with probability p, where the error is drawn randomly from $\{ZI, IZ, ZZ\}$. In addition, each stabilizer measurement outcome is flipped with probability $q = p$. To compare the performance of the decoder for this error model against the iid phase-flip error model described in Definition 2, we cannot simply use the same values of p, as the parameter has different meanings in each model. Instead, we use an effective error rate [3] p_{eff}, which is the marginal probability that a given qubit experiences a phase-flip. In the iid phase-flip error model, $p_{eff} = p$, but in the correlated error model $p_{eff} = 2p - 8p^2/3 + O\left(p^3\right)$. Using the effective error rate as our parameter, we find that behaviour of $p_{\rm th}(N)$ for correlated noise is analogous to the iid phase-flip case, except with a lower sustainable threshold of $p_{\rm sus} \approx 0.8\%$. Supplementary Figure 3 shows the data.

---

[1] A. Kubica and J. Preskill, "Cellular-automaton decoders with provable thresholds for topological codes," *Phys. Rev. Lett.* **123** no. 2, (2019) 020501, `arXiv:1809.10145`.

[2] S. Bravyi and J. Haah, "Quantum self-correction in the 3D cubic code model," *Phys. Rev. Lett.* **111** no. 20, (2013) , `arXiv:1112.3252`.

[3] N. Maskara, A. Kubica, and T. Jochym-O'Connor, "Advantages of versatile neural-network decoding for topological codes," *Phys. Rev. A* **99** no. 5, (2019) 052351, `arXiv:1802.08680`.
